# Supplementary material for: Hybrid Models and Biological Model Reduction with PyDSTool
Source: PLoS Comput Biol. 2012 Aug 9;8(8):e1002628. doi: 10.1371/journal.pcbi.1002628 (PMC3415397; doi:10.1371/journal.pcbi.1002628)
Supplement: Text S4 — Complete source code for the PyDSTool package (version 0.88.120504). Includes API documentation and help files linking to web pages. This file is identical to the current public release on Sourceforge.net. (ZIP) [file pcbi.1002628.s004.zip › PyDSTool/html/PyDSTool.utils-pysrc.html]

xml version="1.0" encoding="ascii"?


PyDSTool.utils


| Home | Trees | Indices | Help | PyDSTool |
| --- | --- | --- | --- | --- |

Package PyDSTool ::
Module utils

[hide private]

[frames] | no frames]

# Source Code for Module PyDSTool.utils

```
  1  """
 
  2      User utilities.
 
  3  """ 
  4  
 
  5  from errors import * 
  6  from common import * 
  7  import Redirector as redirc 
  8  from parseUtils import joinStrs 
  9  
 
 10  from numpy import Inf, NaN, isfinite, less, greater, sometrue, alltrue, \
 
 11       searchsorted, take, argsort, array, swapaxes, asarray, zeros, transpose, \
 
 12       float64, int32, argmin, ndarray, concatenate 
 13  from numpy.linalg import norm 
 14  from scipy.optimize import minpack, zeros 
 15  try: 
 16      newton_meth = minpack.newton 
 17  except AttributeError: 
 18      # newer version of scipy
 
 19      newton_meth = zeros.newton 
 20  import time, sys, os 
 21  import copy 
 22  
 
 23  # --------------------------------------------------------------------
 
 24  
 
 25  # EXPORTS
 
 26  
 
 27  _classes = [] 
 28  
 
 29  _functions = ['intersect', 'remain', 'union', 'cartesianProduct',
 
 30                'makeDataDict', 'makeImplicitFunc', 'orderEventData',
 
 31                'saveObjects', 'loadObjects', 'info', 'compareList',
 
 32                'findClosestArray', 'findClosestPointIndex', 'find',
 
 33                'makeMfileFunction', 'make_RHS_wrap', 'make_Jac_wrap'] 
 34  
 
 35  _mappings = ['_implicitSolveMethods', '_1DimplicitSolveMethods'] 
 36  
 
 37  __all__ = _classes + _functions + _mappings 
 38  
 
 39  
 
 40  rout = redirc.Redirector(redirc.STDOUT) 
 41  rerr = redirc.Redirector(redirc.STDERR) 
 42  
 
 43  ## ------------------------------------------------------------------
 
 44  
 
 45  ## Utility functions
 
 46  
 


47 -def makeMfileFunction(name, argname, defs):


48      """defs is a dictionary of left-hand side -> right-hand side definitions""" 
 49      # writeout file <name>.m
 
 50      mfile = open(name+".m", 'w') 
 51      mfile.write("function %s = %s(%s)\n"%(name,name,argname)) 
 52      for k, v in defs.iteritems(): 
 53          if k != name: 
 54              mfile.write("%s = %s;\n"%(k,v)) 
 55      # now the final definition of tau_recip or inf
 
 56      mfile.write("%s = %s;\n"%(name,defs[name])) 
 57      mfile.write("return\n") 
 58      mfile.close()

 59  
 
 60  
 


61 -def info(x, specName="Contents", offset=1, recurseDepth=1,
 
 62                   recurseDepthLimit=2, _repeatFirstTime=False):


63      """Pretty printer for showing argument lists and dictionary
 
 64      specifications.""" 
 65  
 
 66      if recurseDepth == 1: 
 67          if not _repeatFirstTime: 
 68              # first time through
 
 69              print "Information for " + specName + "\n" 
 70      else: 
 71          print specName + ":", 
 72      if x.__class__ is type: 
 73          return 
 74      if hasattr(x, 'iteritems'): 
 75          x_keys = sortedDictKeys(x) 
 76          if len(x_keys) == 0: 
 77              print "< empty >" 
 78          elif recurseDepth != 1: 
 79              print "" 
 80          for k in x_keys: 
 81              v = x[k] 
 82              kstr = object2str(k) 
 83              basestr = " "*(offset-1) + kstr 
 84              if hasattr(v, 'iteritems'): 
 85                  info(v, basestr, offset+4, recurseDepth+1,
 
 86                               recurseDepthLimit) 
 87              else: 
 88                  vStrList = object2str(v).split(', ') 
 89                  if len(vStrList)==0: 
 90                      vStrList = ['< no information >'] 
 91                  elif len(vStrList)==1 and vStrList[0] == '': 
 92                      vStrList = ['< empty >'] 
 93                  outStrList = [basestr+": "] 
 94                  for i in range(len(vStrList)): 
 95                      if len(vStrList[i] + outStrList[-1]) < 78: 
 96                          outStrList[-1] += ", "*(i>0) + vStrList[i] 
 97                      else: 
 98                          if i>0: 
 99                              if i != len(vStrList): 
100                                  # add trailing comma to previous line
 
101                                  outStrList[-1] += "," 
102                              # start on new line
 
103                              outStrList.append(" "*(len(kstr)+3) + vStrList[i]) 
104                          else: 
105                              # too long for line and string has no commas
 
106                              # could work harder here, but for now, just include
 
107                              # the long line
 
108                              outStrList[-1] += vStrList[i] 
109                  if recurseDepth==1 and len(outStrList)>1: 
110                      # print an extra space between topmost level entries
 
111                      # provided those entries occupy more than one line.
 
112                      print "\n" 
113                  for s in outStrList: 
114                      print s 
115      elif hasattr(x, '__dict__') and recurseDepth <= recurseDepthLimit: 
116          info(x.__dict__, specName, offset, recurseDepth,
 
117                       recurseDepthLimit, True) 
118      else: 
119          xstr = repr(x) 
120          if xstr == '': 
121              xstr = '< no information >' 
122          print xstr

123  
 
124  
 
125  _implicitSolveMethods = ['newton', 'bisect', 'steffe', 'fsolve'] 
126  _1DimplicitSolveMethods = ['newton', 'bisect', 'steffe'] 
127  
 
128  
 


129 -def makeImplicitFunc(f, x0, fprime=None, extrafargs=(), xtolval=1e-8,
 
130                          maxnumiter=100, solmethod='newton', standalone=True):


131      """Builds an implicit function representation of an N-dimensional curve
 
132      specified by (N-1) equations. Thus argument f is a function of 1 variable.
 
133      In the case of the 'fsolve' method, f may have dimension up to N-1.
 
134  
 
135      Available solution methods are: newton, bisect, steffensen, fsolve.
 
136      All methods utilize SciPy's Minpack wrappers to Fortran codes.
 
137  
 
138      Steffenson uses Aitken's Delta-squared convergence acceleration.
 
139      fsolve uses Minpack's hybrd and hybrj algorithms.
 
140  
 
141      Standalone option (True by default) returns regular function. If False,
 
142      an additional argument is added, so as to be compatible as a method
 
143      definition.""" 
144  
 
145      if solmethod == 'bisect': 
146          assert isinstance(x0, _seq_types), \
 
147                 "Invalid type '"+str(type(x0))+"' for x0 = "+str(x0) 
148          assert len(x0) == 2 
149      elif solmethod == 'fsolve': 
150          assert isinstance(x0, (_seq_types, _num_types)), \
 
151                 "Invalid type '"+str(type(x0))+"' for x0 = "+str(x0) 
152      else: 
153          assert isinstance(x0, _num_types), \
 
154                 "Invalid type '"+str(type(x0))+"' for x0 = "+str(x0) 
155  
 
156      # define the functions that could be used
 
157      # scipy signatures use y instead of t, but this naming is consistent
 
158      # with that in the Generator module
 
159      try: 
160          if standalone: 
161              def newton_fn(t): 
162                  rout.start() 
163  ##                rerr.start()
 
164                  res = float(newton_meth(f, x0, args=(t,)+extrafargs, tol=xtolval,
 
165                                       maxiter=maxnumiter, fprime=fprime)) 
166                  rout.stop() 
167  ##                warns = rout.stop()
 
168  ##                rerr.stop()
 
169                  # return {'result': res, 'warnings': warns}
 
170                  return res

171  
 
172              def bisect_fn(t): 
173                  rout.start() 
174  ##                rerr.start()
 
175                  res = minpack.bisection(f, x0[0], x0[1], args=(t,)+extrafargs,
 
176                                        xtol=xtolval, maxiter=maxnumiter) 
177                  rout.stop() 
178  ##                warns = rout.stop()
 
179  ##                rerr.stop()
 
180                  return res 
181  
 
182              def steffe_fn(t): 
183                  rout.start() 
184  ##                rerr.start()
 
185                  res = minpack.fixed_point(f, x0, args=(t,)+extrafargs,
 
186                                             xtol=xtolval, maxiter=maxnumiter) 
187                  rout.stop() 
188  ##                warns = rout.stop()
 
189  ##                rerr.stop()
 
190                  return res 
191  
 
192              def fsolve_fn(t): 
193                  rout.start() 
194  ##                rerr.start()
 
195                  res = minpack.fsolve(f, x0, args=(t,)+extrafargs,
 
196                                        xtol=xtolval, maxfev=maxnumiter,
 
197                                        fprime=fprime) 
198                  rout.stop() 
199  ##                warns = rout.stop()
 
200  ##                rerr.stop()
 
201                  return res 
202          else: 
203              def newton_fn(s, t): 
204                  rout.start() 
205  ##                rerr.start()
 
206                  res = float(newton_meth(f, x0, args=(t,)+extrafargs, tol=xtolval,
 
207                                       maxiter=maxnumiter, fprime=fprime)) 
208                  rout.stop() 
209  ##                warns = rout.stop()
 
210  ##                rerr.stop()
 
211                  return res 
212  
 
213              def bisect_fn(s, t): 
214                  rout.start() 
215  ##                rerr.start()
 
216                  res = minpack.bisection(f, x0[0], x0[1], args=(t,)+extrafargs,
 
217                                        xtol=xtolval, maxiter=maxnumiter) 
218                  rout.stop() 
219  ##                warns = rout.stop()
 
220  ##                rerr.stop()
 
221                  return res 
222  
 
223              def steffe_fn(s, t): 
224                  rout.start() 
225  ##                rerr.start()
 
226                  res = minpack.fixed_point(f, x0, args=(t,)+extrafargs,
 
227                                             xtol=xtolval, maxiter=maxnumiter) 
228                  rout.stop() 
229  ##                warns = rout.stop()
 
230  ##                rerr.stop()
 
231                  return res 
232  
 
233              def fsolve_fn(s, t): 
234                  rout.start() 
235  ##                rerr.start()
 
236                  res = minpack.fsolve(f, x0, args=(t,)+extrafargs,
 
237                                        xtol=xtolval, maxfev=maxnumiter,
 
238                                        fprime=fprime) 
239                  rout.stop() 
240  ##                warns = rout.stop()
 
241  ##                rerr.stop()
 
242                  return res 
243  
 
244      except TypeError, e: 
245          if solmethod == 'bisect': 
246              infostr = " (did you specify a pair for x0?)" 
247          else: 
248              infostr = "" 
249          raise TypeError("Could not create function" +infostr + ": "+str(e)) 
250  
 
251      if solmethod == 'newton': 
252          return newton_fn 
253      elif solmethod == 'bisect': 
254          if fprime is not None: 
255              print "Warning: fprime argument unused for bisection method" 
256          return bisect_fn 
257      elif solmethod == 'steffe': 
258          if fprime is not None: 
259              print "Warning: fprime argument unused for aitken method" 
260          return steffe_fn 
261      elif solmethod == 'fsolve': 
262          return fsolve_fn 
263      else: 
264          raise ValueError("Unrecognized type of implicit function solver") 
265  
 
266  
 


267 -def findClosestPointIndex(pt, target, tol=Inf, in_order=True):


268      """
 
269      Find index of the closest N-dimensional Point in the target N by M array
 
270      or Pointset. Uses norm of order given by the Point
 
271      or Pointset, unless they are inconsistent, in which case an exception is
 
272      raised, or unless they are both arrays, in which case 2-norm is assumed.
 
273  
 
274      With the in_order boolean option (default True), the function will
 
275      attempt to determine the local "direction" of the values and return an
 
276      insertion index that will preserve this ordering. This option is
 
277      incompatible with the tol option (see below).
 
278  
 
279      If the optional tolerance, tol, is given, then an index is returned only
 
280      if the closest distance is within the tolerance. Otherwise, a ValueError
 
281      is raised. This option is incompatible with the in_order option.
 
282      """ 
283      try: 
284          normord = pt._normord 
285      except AttributeError: 
286          normord = 2 
287      try: 
288          if target._normord != normord: 
289              raise ValueError("Incompatible order of norm defined for inputs") 
290      except AttributeError: 
291          pass 
292  
 
293      dists = [norm(pt-x, normord) for x in target] 
294      index = argmin(dists) 
295  
 
296      if in_order: 
297          if index > 0: 
298              lo_off = 1 
299              # insertion offset index
 
300              ins_off = 1 
301              if index < len(target): 
302                  hi_off = 1 
303              else: 
304                  hi_off = 0 
305          else: 
306              lo_off = 0 
307              hi_off = 2 
308              # insertion offset index
 
309              ins_off = 0 
310  
 
311          pta = array([pt]) # extra [] to get compatible shape for concat 
312          dim_range = range(target.shape[1]) 
313          # neighborhood
 
314          nhood = target[index-lo_off:index+hi_off] 
315          if all(ismonotonic(nhood[:,d]) for d in dim_range): 
316              # try inserting at index, otherwise at index+1
 
317              new_nhood = concatenate((nhood[:ins_off], pta, nhood[ins_off:])) 
318              if not all(ismonotonic(new_nhood[:,d]) for d in dim_range): 
319                  ins_off += 1 
320                  index += 1 
321                  new_nhood = concatenate((nhood[:ins_off], pta, nhood[ins_off:])) 
322                  if not all(ismonotonic(new_nhood[:,d]) for d in dim_range): 
323                      raise ValueError("Cannot add point in order, try deactivating the in_order option") 
324  
 
325      if in_order: 
326          return index 
327      else: 
328          if dists[index] < tol: 
329              return index 
330          else: 
331              raise ValueError("No index found within distance tolerance")

332  
 
333  
 


334 -def findClosestArray(input_array, target_array, tol):


335      """
 
336      Find the set of elements in (1D) input_array that are closest to
 
337      elements in target_array.  Record the indices of the elements in
 
338      target_array that are within tolerance, tol, of their closest
 
339      match. Also record the indices of the elements in target_array
 
340      that are outside tolerance, tol, of their match.
 
341  
 
342      For example, given an array of observations with irregular
 
343      observation times along with an array of times of interest, this
 
344      routine can be used to find those observations that are closest to
 
345      the times of interest that are within a given time tolerance.
 
346  
 
347      NOTE: input_array must be sorted! The array, target_array, does not have to be sorted.
 
348  
 
349      Inputs:
 
350        input_array:  a sorted float64 array
 
351        target_array: a float64 array
 
352        tol:          a tolerance
 
353  
 
354      Returns:
 
355        closest_indices:  the array of indices of elements in input_array that are closest to elements in target_array
 
356  
 
357      Author: Gerry Wiener, 2004
 
358      Version 1.0
 
359      """ 
360      # NOT RETURNED IN THIS VERSION:
 
361  #       accept_indices:  the indices of elements in target_array that have a match in input_array within tolerance
 
362  #      reject_indices:  the indices of elements in target_array that do not have a match in input_array within tolerance
 
363  
 
364      input_array_len = len(input_array) 
365      closest_indices = searchsorted(input_array, target_array) # determine the locations of target_array in input_array 
366  #    acc_rej_indices = [-1] * len(target_array)
 
367      curr_tol = [tol] * len(target_array) 
368  
 
369      est_tol = 0.0 
370      for i in xrange(len(target_array)): 
371          best_off = 0          # used to adjust closest_indices[i] for best approximating element in input_array 
372  
 
373          if closest_indices[i] >= input_array_len: 
374              # the value target_array[i] is >= all elements in input_array so check whether it is within tolerance of the last element
 
375              closest_indices[i] = input_array_len - 1 
376              est_tol = target_array[i] - input_array[closest_indices[i]] 
377              if est_tol < curr_tol[i]: 
378                  curr_tol[i] = est_tol 
379  #                acc_rej_indices[i] = i
 
380          elif target_array[i] == input_array[closest_indices[i]]: 
381              # target_array[i] is in input_array
 
382              est_tol = 0.0 
383              curr_tol[i] = 0.0 
384  #            acc_rej_indices[i] = i
 
385          elif closest_indices[i] == 0: 
386              # target_array[i] is <= all elements in input_array
 
387              est_tol = input_array[0] - target_array[i] 
388              if est_tol < curr_tol[i]: 
389                  curr_tol[i] = est_tol 
390  #                acc_rej_indices[i] = i
 
391          else: 
392              # target_array[i] is between input_array[closest_indices[i]-1] and input_array[closest_indices[i]]
 
393              # and closest_indices[i] must be > 0
 
394              top_tol = input_array[closest_indices[i]] - target_array[i] 
395              bot_tol = target_array[i] - input_array[closest_indices[i]-1] 
396              if bot_tol <= top_tol: 
397                  est_tol = bot_tol 
398                  best_off = -1           # this is the only place where best_off != 0 
399              else: 
400                  est_tol = top_tol 
401  
 
402              if est_tol < curr_tol[i]: 
403                  curr_tol[i] = est_tol 
404  #                acc_rej_indices[i] = i
 
405  
 
406          if est_tol <= tol: 
407              closest_indices[i] += best_off 
408  
 
409  #    accept_indices = compress(greater(acc_rej_indices, -1),
 
410  #                                       acc_rej_indices)
 
411  #    reject_indices = compress(equal(acc_rej_indices, -1),
 
412  #                                       arange(len(acc_rej_indices)))
 
413      return closest_indices #, accept_indices, reject_indices)

414  
 
415  
 


416 -def find(x, v, next_largest=1, indices=None):


417      """Returns the index into the 1D array x corresponding to the
 
418      element of x that is either equal to v or the nearest to
 
419      v. x is assumed to contain unique elements.
 
420  
 
421      if v is outside the range of values in x then the index of the
 
422      smallest or largest element of x is returned.
 
423  
 
424      If next_largest == 1 then the nearest element taken is the next
 
425      largest, otherwise if next_largest == 0 then the next smallest
 
426      is taken.
 
427  
 
428      The optional argument indices speeds up multiple calls to this
 
429      function if you have pre-calculated indices=argsort(x).
 
430      """ 
431      if indices is None: 
432          indices=argsort(x) 
433      xs=take(x, indices, axis=0) 
434      assert next_largest in [0,1], "next_largest must be 0 or 1" 
435      eqmask=(xs==v).tolist() 
436      try: 
437          ix = eqmask.index(1) 
438      except ValueError: 
439          if next_largest: 
440              mask=(xs<v).tolist() 
441          else: 
442              mask=(xs>v).tolist() 
443          try: 
444              ix=min([max([0,mask.index(1-next_largest)+next_largest-1]),len(mask)-1]) 
445          except ValueError: 
446              ix = 0+next_largest-1 
447      return indices[ix]

448  
 
449  
 


450 -def orderEventData(edict, evnames=None, nonames=False, bytime=False):


451      """Time-order event data dictionary items.
 
452  
 
453      Returns time-ordered list of (eventname, time) tuples.
 
454  
 
455      If 'evnames' argument included, this restricts output to only the named
 
456        events.
 
457      The 'nonames' flag (default False) forces routine to return only the event
 
458        times, with no associated event names.
 
459      The 'bytime' flag (default False) only works with nonames=False and returns
 
460        the list in (time, eventname) order.
 
461      """ 
462  
 
463      if evnames is None: 
464          evnames = edict.keys() 
465      else: 
466          assert remain(evnames, edict.keys()) == [], "Invalid event names passed" 
467      # put times as first tuple entry of etuplelist
 
468      if nonames: 
469          alltlist = [] 
470          for (evname,tlist) in edict.items(): 
471              if evname in evnames: 
472                  alltlist.extend(tlist) 
473          alltlist.sort() 
474          return alltlist 
475      else: 
476          etuplelist = [] 
477          for (evname,tlist) in edict.items(): 
478              if evname in evnames: 
479                  etuplelist.extend([(t,evname) for t in tlist]) 
480          # sort by times
 
481          etuplelist.sort() 
482          if bytime: 
483              return etuplelist 
484          else: 
485              # swap back to get event names as first tuple entry
 
486              return [(evname,t) for (t,evname) in etuplelist]

487  
 
488  ## ------------------------------------------------------------
 
489  ## Generator wrapping utilities
 
490  
 


491 -def make_RHS_wrap(gen, xdict_base, x0_names, use_gen_params=False, overflow_penalty=1e4):


492      """Return function wrapping Generator argument gen's RHS function,
 
493      but restricting input and output dimensions to those specified by
 
494      x0_names. All other variable values will be given by those in xdict_base.
 
495      In case of overflow or ValueError during a call to the wrapped function,
 
496      an overflow penalty will be used for the returned values (default 1e4).
 
497  
 
498      if use_gen_params flag is set (default False)
 
499      then:
 
500        Return function has signature Rhs_wrap(x,t)
 
501        and takes an array or list of x state variable values and scalar t,
 
502        returning an array type of length len(x). The Generator's current param
 
503        values (at call time) will be used.
 
504      else:
 
505        Return function has signature Rhs_wrap(x,t,pdict)
 
506        and takes an array or list of x state variable values, scalar t, and a
 
507        dictionary of parameters for the Generator, returning an array type of
 
508        length len(x).
 
509  
 
510      NB: xdict_base will be copied as it will be updated in the wrapped
 
511      function.""" 
512      var_ix_map = invertMap(gen.funcspec.vars) 
513      x0_names.sort()   # ensures sorted 
514      x0_ixs = [var_ix_map[xname] for xname in x0_names] 
515      dim = len(x0_names) 
516      xdict = xdict_base.copy() 
517      if use_gen_params: 
518          def Rhs_wrap(x, t): 
519              xdict.update(dict(zip(x0_names, x))) 
520              try: 
521                  return take(gen.Rhs(t, xdict, gen.pars), x0_ixs) 
522              except (OverflowError, ValueError): 
523                  return array([overflow_penalty]*dim)

524  
 
525      else: 
526          def Rhs_wrap(x, t, pdict): 
527              xdict.update(dict(zip(x0_names, x))) 
528              try: 
529                  return take(gen.Rhs(t, xdict, pdict), x0_ixs) 
530              except (OverflowError, ValueError): 
531                  return array([overflow_penalty]*dim) 
532  
 
533      return Rhs_wrap 
534  
 
535  
 


536 -def make_Jac_wrap(gen, xdict_base, x0_names, use_gen_params=False, overflow_penalty=1e4):


537      """Return function wrapping Generator argument gen's Jacobian function,
 
538      but restricting input and output dimensions to those specified by
 
539      x0_names. All other variable values will be given by those in xdict_base.
 
540      In case of overflow or ValueError during a call to the wrapped function,
 
541      an overflow penalty will be used for the returned values (default 1e4).
 
542  
 
543      if use_gen_params flag is set (default False)
 
544      then:
 
545        Return function Jac_wrap(x,t) takes an array or list of x variable
 
546        values and scalar t, returning a 2D array type of size len(x) by len(x).
 
547        The Generator's current param values (at call time) will be used.
 
548      else:
 
549        Return function Jac_wrap(x,t,pdict) takes an array or list of x variable
 
550        values, scalar t, and a dictionary of parameters for the Generator,
 
551        returning a 2D array type of size len(x) by len(x).
 
552  
 
553      NB: xdict_base will be copied as it will be updated in the wrapped
 
554      function.""" 
555      if not gen.haveJacobian(): 
556          raise ValueError("Jacobian not defined") 
557      var_ix_map = invertMap(gen.funcspec.vars) 
558      x0_names.sort()   # ensures sorted 
559      x0_ixs = [var_ix_map[xname] for xname in x0_names] 
560      dim = len(x0_names) 
561      xdict = xdict_base.copy() 
562      if use_gen_params: 
563          def Jac_wrap(x, t): 
564              xdict.update(dict(zip(x0_names, x))) 
565              try: 
566                  return take(take(gen.Jacobian(t, xdict, gen.pars), x0_ixs,0), x0_ixs,1) 
567              except (OverflowError, ValueError): 
568                  return array([overflow_penalty]*dim)

569      else: 
570          def Jac_wrap(x, t, pdict): 
571              xdict.update(dict(zip(x0_names, x))) 
572              try: 
573                  return take(take(gen.Jacobian(t, xdict, pdict), x0_ixs,0), x0_ixs,1) 
574              except (OverflowError, ValueError): 
575                  return array([overflow_penalty]*dim) 
576  
 
577      return Jac_wrap 
578  
 
579  
 
580  
 
581  ## ------------------------------------------------------------
 
582  
 


583 -def saveObjects(objlist, filename, force=False):


584      """Store PyDSTool objects to file. Argument should be a tuple or list,
 
585      but if a singleton non-sequence object X is given then it will be
 
586      saved as a list [ X ].
 
587  
 
588      Some PyDSTool objects will not save using this function, and will complain
 
589      about attributes that do not have definitions in __main__.
 
590      """ 
591  
 
592      # passing protocol = -1 to pickle means it uses highest available
 
593      # protocol (e.g. binary format)
 
594      if not force: 
595          if os.path.isfile(filename): 
596              raise ValueError("File '" + filename + "' already exists") 
597      pklfile = file(filename, 'wb') 
598      # Win32 only: in call to pickle.dump ...
 
599      # DO NOT use binary option (or HIGHESTPROTOCOL) because
 
600      # IEE754 special values are not UNpickled correctly in Win32
 
601      # (you'll see no exception raised). This is a known bug
 
602      # and fixedpickle.py is a work-around. (June 2005)
 
603      if os.name == 'nt': 
604          opt = None 
605      else: 
606          opt = 0 
607      if not isinstance(objlist, list): 
608          objlist=[objlist] 
609      for obj in objlist: 
610          try: 
611              pickle.dump(obj, pklfile, opt) 
612          except: 
613              if hasattr(obj, 'name'): 
614                  print "Failed to save '%s'"%obj.name 
615              else: 
616                  print "Failed to save object '%s'"%str(obj) 
617              raise 
618      pklfile.close()

619  
 
620  
 
621  
 


622 -def loadObjects(filename, namelist=None):


623      """Retrieve PyDSTool objects from file. Returns list of objects
 
624      unless namelist option is given as a singleton string name.
 
625      Also, if only one object X was stored, it will be returned as [X],
 
626      and thus you will have to index the returned list with 0 to get X itself.
 
627  
 
628      Optional namelist argument selects objects to return by name,
 
629      provided that the objects have name fields (otherwise they are ignored).
 
630      If namelist is a single string name then a single object is returned.
 
631      """ 
632  
 
633      # Since names are not intended to be unique in PyDSTool, the while
 
634      # loop always goes to the end of the file, and pulls out *all*
 
635      # occurrences of the names.
 
636      if not os.path.isfile(filename): 
637          raise ValueError("File '" + filename + "' not found") 
638      if namelist is None: 
639          namelist = [] 
640      was_singleton_name = isinstance(namelist, str) 
641      if not isinstance(namelist, list): 
642          if was_singleton_name: 
643              namelist = [copy.copy(namelist)] 
644          else: 
645              raise TypeError("namelist must be list of strings or singleton string") 
646      if not isUniqueSeq(namelist): 
647          raise ValueError("Names must only appear once in namelist argument") 
648      pklfile = file(filename, 'rb') 
649      if namelist == []: 
650          getall = True 
651      else: 
652          getall = False 
653      objlist = [] 
654      notDone = True 
655      while notDone: 
656          try: 
657              if getall: 
658                  objlist.append(pickle.load(pklfile)) 
659              else: 
660                  tempobj = pickle.load(pklfile) 
661                  if hasattr(tempobj, 'name'): 
662                      if tempobj.name in namelist: 
663                          objlist.append(tempobj) 
664          except EOFError: 
665              notDone = False 
666          except: 
667              print "Error in un-pickling:" 
668              print "Was the object created with an old version of PyDSTool?" 
669              pklfile.close() 
670              raise 
671      pklfile.close() 
672      if objlist == []: 
673          if getall: 
674              print "No objects found in file" 
675          else: 
676              print "No named objects found in file" 
677      if was_singleton_name: 
678          return objlist[0] 
679      else: 
680          return objlist

681  
 
682  
 


683 -def intersect(a, b):


684      """Find intersection of two lists, sequences, etc.
 
685      Returns a list that includes repetitions if they occur in the inputs.""" 
686      return filter(lambda e : e in b, a)

687  
 


688 -def union(a, b):


689      """Find union of two lists, sequences, etc.
 
690      Returns a list that includes repetitions if they occur in the input lists.
 
691      """ 
692      return list(a)+list(b)

693  
 


694 -def remain(a, b):


695      """Find remainder of two lists, sequences, etc., after intersection.
 
696      Returns a list that includes repetitions if they occur in the inputs.""" 
697      return filter(lambda e : e not in b, a)

698  
 


699 -def compareList(a, b):


700      """Compare elements of lists, ignoring order (like sets).""" 
701      return len(intersect(a,b))==len(a)==len(b)

702  
 


703 -def cartesianProduct(a, b):


704      """Returns the cartesian product of the sequences.""" 
705      ret = [] 
706      for i in a: 
707          ret.extend([(i, j) for j in b]) 
708      return ret

709  
 
710  # deprecated
 


711 -def makeDataDict(fieldnames, fieldvalues):


712      """Zip arrays of field names and values into a dictionary.
 
713      For instance, to use in Generator initialization arguments.
 
714  
 
715      Deprecated as of v0.89.""" 
716      if isinstance(fieldvalues, ndarray): 
717          return dict(zip(fieldnames, [a.tolist() for a in fieldvalues])) 
718      else: 
719          return dict(zip(fieldnames, fieldvalues))

720
```

  


| Home | Trees | Indices | Help | PyDSTool |
| --- | --- | --- | --- | --- |

|  |  |
| --- | --- |
| Generated by Epydoc 3.0.1 on Fri May 4 15:24:15 2012 | http://epydoc.sourceforge.net |
